# Short‐Term Exposure to Fine Particulate Matter (PM_2.5_), Cause Specific‐Mortality, and High‐Risk Populations: A Nationwide Time‐Stratified Case‐Crossover Study

**DOI:** 10.1029/2024GH001214

**Published:** 2025-09-26

**Authors:** Seoyeong Ahn, Jieun Oh, Hyewon Yun, Harin Min, Yejin Kim, Cinoo Kang, Sojin An, Ayoung Kim, Dohoon Kwon, Jinah Park, Whanhee Lee

**Affiliations:** ^1^ Department of Information Convergence Engineering College of Information and Biomedical Engineering Pusan National University Yangsan South Korea; ^2^ The Environmental Health Center for Climate Change Pusan National University Yangsan South Korea; ^3^ Department of Public Health Sciences Graduate School of Public Health Seoul National University Seoul South Korea; ^4^ Graduate School of Data Science Pusan National University Busan South Korea; ^5^ School of Biomedical Convergence Engineering College of Information and Biomedical Engineering Pusan National University Yangsan South Korea

## Abstract

Numerous studies have reported that short‐term exposure to fine particulate matter (PM_2.5_) is associated with mortality risk; however, results on high‐risk populations and regions have been mixed. This study performed a nationwide time‐stratified case‐crossover study to assess the association between short‐term PM_2.5_ and mortality in South Korea (2015–2019) by each cause of death and age group. A machine‐learning ensemble PM_2.5_ prediction model was used to cover unmonitored districts. We estimated the excess mortality and Years of Life Lost (YLL) attributable to PM_2.5_ and non‐compliance with the 2021 WHO guidelines (>15 μg/m^3^). We examined the effect modifications by district‐level accessibility to green spaces and medical facilities in the living sphere. In the total population, PM_2.5_ was positively associated with mortality for non‐accidental causes (OR: 1.008 with 95% CI: 1.006–1.010), circulatory diseases (1.007, 95% CI: 1.003–1.011), and respiratory diseases (1.007, 95% CI: 1.001–1.013). Based on the point estimates, the association was generally greater in younger age groups (0–59 or 60–69 years) than in older age groups (70–80 and 80 years or older), and this pattern was pronounced in mortality for cerebrovascular diseases and pneumonia. The excess mortality fraction and YLL due to non‐compliance with WHO guidelines were 0.80% and 186,808.52 years. Our findings suggest high risk populations and benefits for establishing stricter PM_2.5_ standards and action plans.

## Plain Language Summary

Despite numerous studies identifying the hazardous impacts of fine particulate matter (PM_2.5_) and mortality, the high‐risk individuals and regions to the PM_2.5_‐mortality risk considering multiple risk factors are still uncertain. In this study including 1,270,499 mortality cases (study period: 2015–2019), we found high‐risk age groups to PM_2.5_ and mortality: younger age populations (0–59 years or 60–69 years), especially with potential risk factors related to cerebrovascular diseases or pneumonia. We also revealed a weak tendency that districts with better accessibility to parks in the living sphere had a higher PM_2.5_ risk of mortality. Further, we estimated that among all deaths, the excess proportion of mortality and years of premature death (i.e., years of life lost) attributable to non‐compliance with the current WHO air quality guidelines (>15 μg/m^3^) was 0.80% and 186,808.52 years during the study period.

## Key Points

- This study evaluated the nationwide risk estimates of fine particulate matter (PM_2.5_) on mortality in South Korea
- We found high‐risk age groups to PM_2.5_ and mortality: younger age populations (0–59 years or 60–69 years)
- The excess mortality fraction and Years of Life Lost due to non‐compliance with the 2021 World Health Organization air quality guidelines were 0.80% and 186,808.52 years

## 1 Introduction

Ambient fine particulate matter (PM_2.5_) has been regarded as a crucial public health concern, and the World Health Organization (WHO) reported in 2019 that 4.2 million people die prematurely each year due to ambient air pollution (Shaddick et al., 2020). Numerous epidemiological studies have identified the hazardous impacts of PM_2.5_ on mortality and morbidities since the 1990s (Bell et al., 2005; Liu et al., 2019) and several recent studies revealed that PM_2.5_ might have a causal relationship with mortality even at low concentrations (Wei et al., 2020; Wu et al., 2020).

Despite these numerous studies, several important knowledge gaps regarding high‐risk populations still exist. First, most existing studies reported that people aged above 65 or 75 years, socially marginalized persons, and individuals with underlying diseases are high‐risk populations for PM_2.5_‐related mortality risks (Lipfert, 2017; Ma et al., 2023). However, evidence for high‐risk groups regarding younger populations with potential risk factors or regional characteristics on medical infrastructure was highly limited. Second, many previous studies investigated populations residing in well‐monitored urban areas (Di, Wang, et al., 2017), and it indicates that previous results might provide limited information on less‐monitored areas (i.e., selection biases). Third, previous studies had limitations in assessing the potential effect modifications by regional characteristics on the association between PM_2.5_ and mortality.

To address these gaps in knowledge, we performed a nationwide time‐stratified case‐crossover study using the national mortality data (2015–2019) in South Korea. In particular, to reveal various high‐risk populations, five age groups, three major and six specific causes of death, and four district‐level indicators covering urbanicity and medical infrastructure were examined. To cover unmonitored areas, we used a machine learning‐based ensemble air pollution prediction model with excellent performance.

## 2 Methods

### 2.1 Study Populations

We collected all individual‐level mortality cases registered in Statistics Korea from 1st January 2015 to 31st December 2019 in the 247 districts of South Korea. To evaluate the age‐specific association between PM_2.5_ and mortality, we divided ages at death into five categories: total (all ages), individuals aged 0–59 years, 60–69 years, 70–79 years, and those aged 80 years or older.

Further, we investigated a total of nine causes of death based on the International Classification of Diseases 10th Revision (ICD‐10). First, we defined three major causes of death: (a) non‐accidental causes (ICD‐10: A00‐R99), (b) diseases of the circulatory system (I00‐I99), and (c) diseases of the respiratory system (J00‐J99). Second, six specific causes of death were also examined: ischemic heart diseases (I20–I25), cerebrovascular diseases (I60–69), other circulatory diseases (I00–I15, I26–52, and I79–I99), pneumonia (J12–J18), chronic lower respiratory diseases (J40–J47), and other respiratory diseases (J00–J11, J30–J39, and J60–J99). Detailed information on the non‐accidental causes of death is provided in the Supporting Information S1 (“1. Causes of death ‐ non‐accidental causes of death”).

Due to the identification issue, Statistics Korea only provides the specific causes of death based on the three‐character “categories” of the diagnosis (e.g., ischemic heart diseases [I20–I25]). Thus, we could not address more specific causes of death, like heart failure or asthma, because these are “single” diseases that could not be defined by the three‐character categories. For example, cardiac arrest and heart failure were included in the category of other circulatory diseases, and influenza and acute respiratory infections were included in other respiratory diseases.

### 2.2 Study Design

We adopted a time‐stratified case‐crossover design and defined a case day as the date of death. For each case day, we identified matched control days with the same day of the week within the same month and same year. This time‐stratified self‐matching controlled for confounding variables that do not change substantially in a month, such as age, sex, weight, diet, and district‐level variables like population compositions, regional income status, and accessibility to facilities (Wei et al., 2019). The time‐stratified matching also controlled for potential confounding that varies across weekdays and weekends, seasonality, and long‐term trends of PM_2.5_ and outcomes. This approach has been widely used to estimate the risks of short‐term environmental stressors on acute health outcomes (Park et al., 2024; Sun et al., 2021; Wei et al., 2019).

### 2.3 Air Pollution and Environmental Data

We collected nationwide district‐level 24‐hr average modeled daily PM_2.5_ and nitrogen dioxide (NO_2_) (2015–2019; μg/m^3^) by machine‐learning ensemble prediction models developed by the AiMS‐CREATE team. This modeling data was used in previously published studies (Kim et al., 2024; Min et al., 2024). The ensemble model incorporates three machine‐learning algorithms (random forest, extreme gradient boosting, and deep neural network) with 1 km^2^ spatial resolution. Detailed information on the model is provided in the Supporting Information S1 (“2. Air pollution prediction models”). The daily PM_2.5_ and NO_2_ prediction models showed excellent performances: cross‐validated R^2^ values of 0.94 and 0.95 (Table S1 in Supporting Information S1).

Because the national mortality data only provided district‐level residential addresses (“*si*/*gun*/*gu*”), daily concentration predictions at 1 km^2^ were aggregated to each district by averaging the predictions at grid cells with centroid points inside the boundary of that district. Lastly, we used the average PM_2.5_ on the same and previous days of each mortality (lag 0–1) as the main exposure based on the Akaike Information Criterion (AIC; i.e., lag 0–1 showed the lowest average AIC values across all populations, compared to lag 0–2 and lag 0–3), consistent with previous studies on the health impacts of short‐term PM_2.5_ (Di, Dai, et al., 2017; Sun et al., 2024; Wei et al., 2019).

We also collected meteorological variables from the ERA‐5 Land global reanalysis data set, and these variables include 24‐hr average 2m air temperature (K), relative humidity (%), and precipitation (m) (Park et al., 2024).

### 2.4 District Indicators

To examine the potential spatial differences in PM_2.5_‐mortality risk according to regional characteristics, we collected data on four district‐level indicators covering urbanicity, vegetation, and levels of medical accessibility: population density (persons per km^2^), distance to parks in the living sphere, the number of beds in hospitals per 1,000 persons (hereafter, the number of hospital beds) and distance to emergency medical facilities. Detailed information on the data sources and explanations of these district indicators is reported in the Supporting Information S1 (“3. District‐level indicators”). We used average values of each indicator and classified all indicators into three categories (high, middle, and low) based on their tertiles (Byun et al., 2024).

### 2.5 Statistical Analysis

In the main analysis, we created a time‐stratified case‐crossover data set for each cause of death and age group (a total of 45 combinations; nine causes of death with five age categories). For each data set, we fitted a conditional logistic regression to estimate the association between short‐term PM_2.5_ and mortality. To control for potential confounders, we adjusted for relative humidity, precipitation, and moving‐averaged temperatures (lag 0–3) using a natural cubic spline with six degrees of freedom (Liu et al., 2019). We calculated the odds ratio (OR) for a 10 μg/m^3^ increase in PM_2.5_ to measure the association between PM_2.5_ and mortality. To assess any potential deviations from linearity in the concentration‐response curves, we performed an additional analysis by replacing the linear term of PM_2.5_ with a natural spline function with three equally distributed internal knots.

We performed additional analyses to examine whether the estimated PM_2.5_‐mortality risks were modified by district‐level characteristics and whether the modifications differed by cause of death and age group. For each case‐crossover data set, we added an interaction term between PM_2.5_ and the district indicator (categorized into tertiles) and repeated the main analysis. We calculated ORs for each district indicator category (high/middle/low).

Lastly, we performed several sensitivity analyses to examine whether our results are robust to different modeling specifications regarding lag days and confounder adjustments. In all statistical analyses, R software (version 4.2.1) with packages “*dlnm*,” “*tsModel,*” and “*splines*” are used.

### 2.6 Excess Mortality and Years of Life Lost Attributable to PM_2.5_

Risk estimates for PM_2.5_ (ORs) were translated into excess mortality and YLL (years of life lost from mortality) attributable to PM_2.5_, to demonstrate the change in mortality burden due to PM_2.5_ exposures. Detailed calculation procedures of the excess mortality and YLL are reported in the Supporting Information S1 (“4. The excess mortality and Years of life lost from mortality attributable to PM_2.5_”). Briefly, we first calculated the daily excess mortality numbers and YLL attributable to PM_2.5_ using daily PM_2.5_ concentrations and estimated ORs. Then, the sum of daily attributable deaths and YLL represents the total excess number of deaths and YLLs attributable to PM_2.5_ during the study period. The total excess number of deaths ratio with the total number of deaths provides the total excess fraction of mortality attributable to PM_2.5_. We used Monte Carlo simulations to calculate the confidence intervals of each estimate, using 1,000 replicates (Lee et al., 2022). In addition, to assess the mortality burden associated with non‐compliance with the 2021 WHO guidelines regarding PM_2.5_ (daily average PM_2.5_ > 15 μg/m^3^), we calculated attributable deaths and YLL only for daily PM_2.5_ concentrations of 15 μg/m^3^ or higher (i.e., the excess mortality fraction (%) computed as the sum of the daily excess death counts due to daily PM_2.5_ above >15μg/m^3^ divided by the total mortality counts).

## 3 Results

**Figure 1:**
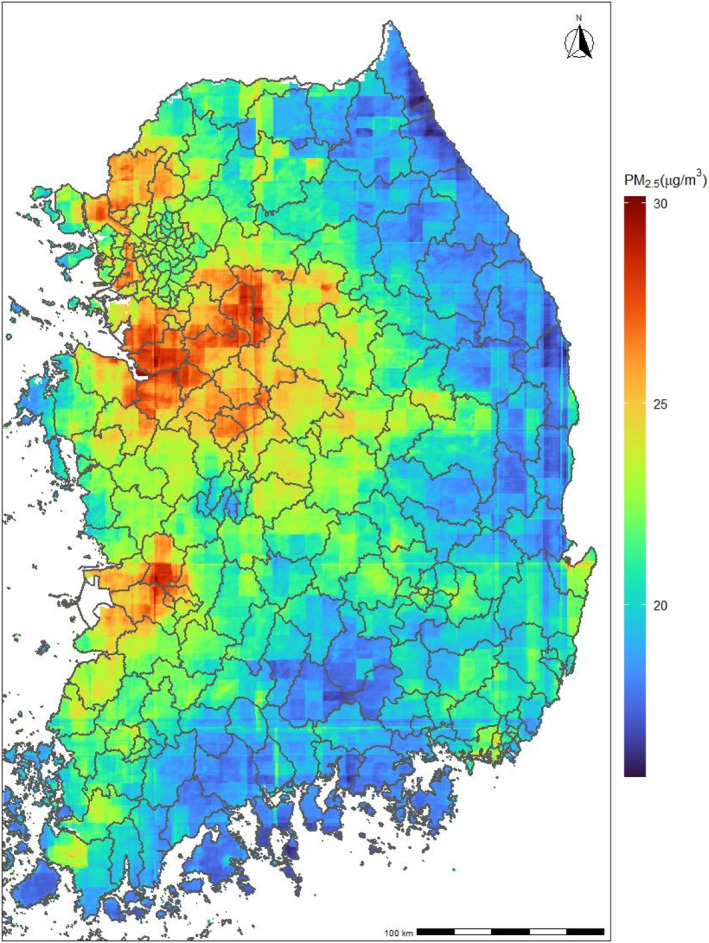
Geographical distributions of the annual averages of daily average PM_2.5_ (μg/m^3^) in South Korea from 2015 through 2019.

Table 1 provides descriptive statistics of mortality. A total of 1,270,499 mortality cases were included during the study period (2015–2019). Among them, 23.5% of cases were from cardiovascular disease, and 12.7% were from respiratory disease. Around 47.2% of non‐accidental deaths were from people aged 80 years or older, and individuals aged 0–59 and 60–69 years consisted of 14.2% and 13.1%. Figure 1 displays the average spatial distribution of PM_2.5_ in South Korea during the study period (25.04 μg/m^3^ average across all regions).

**Table 1:** Descriptive Statistics on Mortality Data (2015–2019)

Causes of death	Age categories	Case	(%)
Non‐accidental			
	Total	1,270,499	100.0
	0‐59 years	180,893	14.2
	60‐69 years	166,658	13.1
	70‐79 years	323,411	25.5
	80 years and older	599,336	47.2
Circulatory disease			
	Total	298,838	23.5
	0‐59 years	33,493	2.6
	60‐69 years	31,457	2.5
	70‐79 years	74,205	5.8
	80 years and older	159,643	12.6
Respiratory disease			
	Total	161,059	12.7
	0‐59 years	6,396	0.5
	60‐69 years	11,741	0.9
	70‐79 years	39,628	3.1
	80 years and older	103,271	8.1

*Note*. Denominator of % was the total non‐accidental deaths.

**Figure 2:**
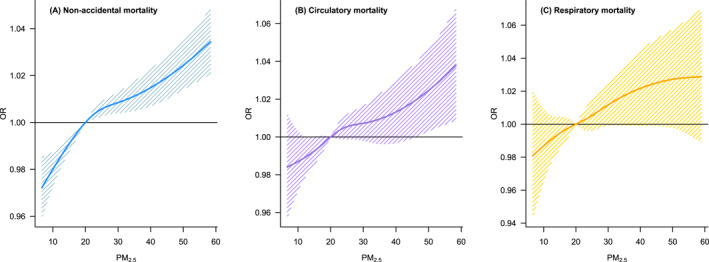
Nonlinear association between short‐term PM_2.5_ exposure and mortality by cause of death. OR: odds ratio.

**Figure 3:**
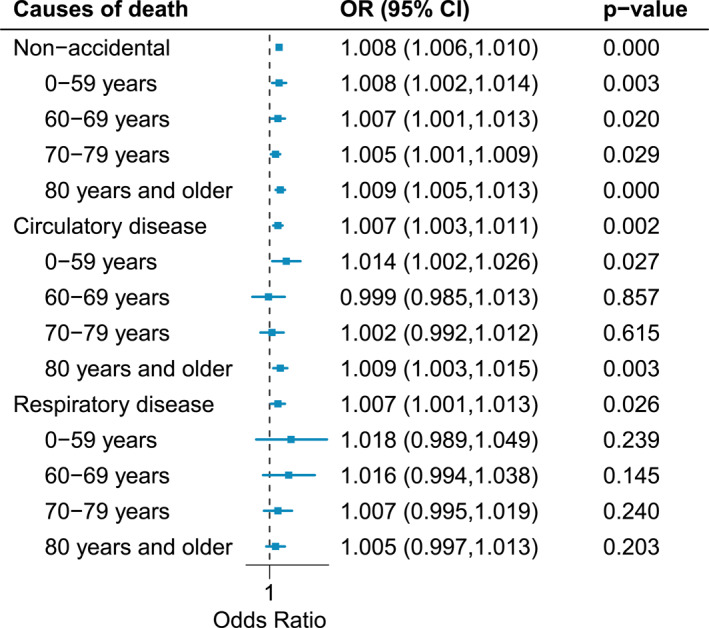
Association between short‐term PM_2.5_ exposure and mortality by cause of death and age group. OR: odds ratio for a 10 μg/m^3^ increase in PM_2.5_, *P*‐value: *P*‐value of OR (H_0_: OR is one).

Figure 2 shows flexible PM_2.5_‐mortality curves, and all causes showed a linear relationship with PM_2.5_. Figure 3 shows the cause‐specific associations between PM_2.5_ and mortality in the total population and by age group. In the total population, PM_2.5_ had positive associations with mortality for non‐accidental causes (OR: 1.008 with 95% CI: 1.006–1.010), circulatory diseases (1.007 with 95% CI: 1.003–1.011), and respiratory diseases (1.007 with 95% CI: 1.001–1.013). The associations were heterogeneous by age group. For non‐accidental death, individuals aged 0–69 years showed slightly higher PM_2.5_ risks compared to those aged 70–79 years based on point estimates, and the risk was the highest in people aged 80 years or older. This pattern by age became more prominent in circulatory and respiratory mortalities.

The association between PM_2.5_ and circulatory mortality was the strongest in people aged 0–59 years (OR: 1.014 with 95% CI: 1.002–1.026). Whereas, the association was not evident in the age groups 60–79 years. Further, although the association between PM_2.5_ and respiratory death was not statistically evident across all age groups, the association with PM_2.5_ was higher in younger age groups (0–59 and 60–69 years) than in older age groups based on the point estimates. The age patterns were generally consistent with specific causes of death (Table S2 in Supporting Information S1): individuals aged 0–59 or 60–69 years showed higher PM_2.5_ risks of mortality for cerebrovascular diseases and pneumonia. Further, males showed slightly higher ORs than females, and the age pattern was more prominent in males than in females (Figure S1 in Supporting Information S1).

**Figure 4:**
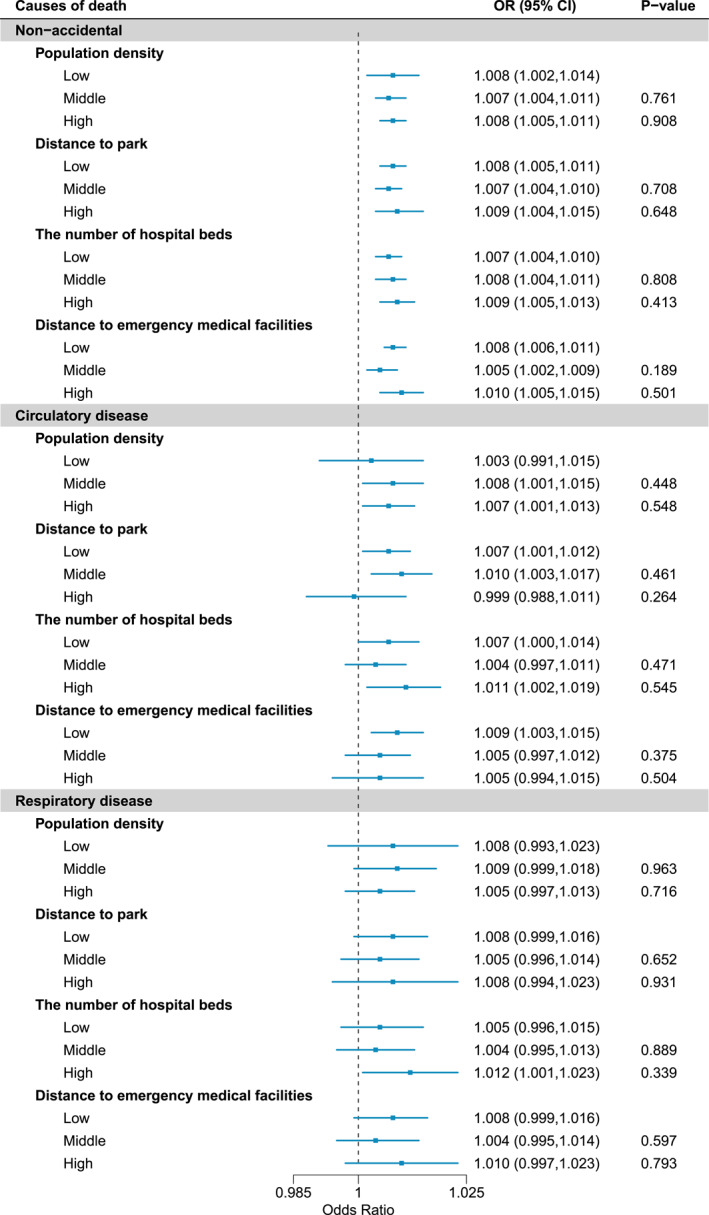
Effect modifications by district‐level indicators in the PM_2.5_‐mortality risk in the total population. OR: odds ratio for a 10 μg/m^3^ increase in PM_2.5_, *P*‐value: *P*‐value of OR (H_0_: OR is one). *P*‐values indicate the statistical test for differences in middle or high levels compared to low levels (*H*_0_: no difference in OR estimates among categories).

Figure 4 presents the associations between PM_2.5_ and cause‐specific mortalities by district indicator categories in the total population. P‐values in Figure 4 indicate the statistical test for differences in middle or high levels compared to low levels. Across all causes and indicator categories, we could not find strong statistical evidence for the effect modifications by district‐level characteristics. A weak modification by distance to parks in the living space was observed in non‐accidental mortality: the district with the medium‐level distance to emergency medical facilities showed a lower association between PM_2.5_ and non‐accidental mortality risk (P‐value: 0.189). The modification by distance to parks and emergency medical facilities was observed in mortality for pneumonia (Table S3 in Supporting Information S1). However, we could not find an obvious difference between the sexes (Figure S2 in Supporting Information S1).

The estimated excess mortality and YLL attributable to short‐term PM_2.5_ are reported in Table 2. During the entire study period (5 years), the excess mortality fractions attributable to the whole range of PM_2.5_ were 1.88% (95% CI: 1.37%–2.4%) for non‐accidental mortality, 1.71% (0.64%–2.80%) for circulatory mortality, 1.67% (0.22%–3.15%) for respiratory mortality, in the total population. For days of non‐compliance with WHO guidelines for PM_2.5_, the excess death fractions were 0.80% (0.58%–1.03%) in non‐accidental mortality, 0.73% (0.27%–1.20%) for circulatory mortality, 0.72% (0.09%–1.36%) for respiratory mortality, and these accounted for around 40% of the total excess mortality due to PM_2.5_. The excess mortality fraction was generally higher in individuals aged 0–59 years compared to the other age groups, except for non‐accidental death, showing the excess mortality fractions for individuals aged 0–59 years (2.05%) and 80 years or older (2.26%). Table S4 in Supporting Information S1 shows the absolute number of excess deaths attributable to PM_2.5_, and the excess number of deaths was the highest in those aged 80 years or older because of the large number of deaths. We estimated the excess YLL attributable to the entire range of PM_2.5_: 436,185.64 years (95% CI: 317,717.29 years‐557,929.54 years), and the excess YLL due to non‐compliance with WHO guidelines was 186,808.52 years (135,948.67 years–239,142.42 years) for non‐accidental mortality in the total population.

**Table 2:** Excess Death Fractions and YLL (Year of Life Lost From Mortality) Attributable to Ambient Short‐Term Exposure to PM_2.5_ (Lag 0–1) for All‐Cause Mortality

Cause of death	Age group	All‐range of PM_2.5_		WHO guideline compliance	
		Excess deaths (%)	Excess YLL (years)	Excess deaths (%)	Excess YLL (years)
Non‐accidental	Total	1.88 (1.37–2.4)	436185.64 (317717.29–557929.54)	0.80 (0.58–1.03)	186808.52 (135948.67–239142.42)
	0‐59 years	2.05 (0.70–3.42)	150861.73 (51788.94–251858.25)	0.88 (0.30–1.48)	65090.14 (22287.13–108872.82)
	60‐69 years	1.69 (0.28–3.12)	71716.95 (11702.84–132861.99)	0.72 (0.12–1.33)	30562.45 (4973.22–56726.90)
	70‐79 years	1.17 (0.13–2.22)	62328.35 (6979.67–118928.01)	0.50 (0.06–0.95)	26603.87 (2972.89–50832.79)
	80 years and older	2.26 (1.52–3.02)	140486.83 (94546.93–187591.73)	0.97 (0.65–1.29)	60055.47 (40364.21–80283.70)
Circulatory disease	Total	1.71 (0.64–2.80)	85459.91 (32128.67–139975.88)	0.73 (0.27–1.20)	36712.07 (13773.76–60223.55)
	0‐59 years	3.53 (0.44–6.62)	46665.79 (5801.31–87623.09)	1.53 (0.19–2.89)	20230.67 (2499.56–38141.29)
	60‐69 years	−0.29 (−3.66–3.07)	−2356.72 (−29289.62–24600.59)	−0.12 (−1.56–1.32)	−992.55 (−12462.99–10583.39)
	70‐79 years	0.58 (−1.64–2.82)	7007.53 (−19952.60–34278.64)	0.25 (−0.70–1.21)	3017.57 (−8529.48–14764.02)
	80 years and older	2.22 (0.77–3.70)	36740.13 (12766.56–61154.69)	0.95 (0.33–1.59)	15722.53 (5448.12–26224.50)
Respiratory disease	Total	1.67 (0.22–3.15)	37622.17 (4851.12–70997.48)	0.72 (0.09–1.36)	16202.60 (2083.16–30635.00)
	0‐59 years	4.30 (−2.93–11.26)	10840.20 (−7396.92–28407.78)	1.89 (−1.27–4.99)	4769.84 (−3202.13–12587.27)
	60‐69 years	3.98 (−1.37–9.22)	11752.74 (−4051.10–27258.54)	1.72 (−0.59–4.01)	5085.56 (−1732.06–11868.20)
	70‐79 years	1.79 (−1.19–4.78)	11552.12 (−7642.48–30823.00)	0.77 (−0.51–2.06)	4976.96 (−3269.52–13319.40)
	80 years and older	1.19 (−0.63–3.04)	12660.15 (−6656.90–32265.86)	0.51 (−0.27–1.31)	5441.65 (−2849.74–13894.72)

Lastly, in the sensitivity analyses (Table S5 in Supporting Information S1), our risk estimates in the total population and patterns by age groups remained consistent with different PM_2.5_ lag days, degree of freedoms of the temperature spline function, temperature lag days, and NO_2_ adjustment (i.e., a double‐pollutant model).

## 4 Discussion

This study examined the nationwide associations between short‐term exposure to PM_2.5_ and cause‐specific mortality and high‐risk populations in South Korea, based on over a million mortality cases and the PM_2.5_ prediction model with excellent performance. The major finding of this study is that the association between PM_2.5_ and mortality was generally prominent in younger populations (aged 0–59 years or 60–69 years) compared to older populations, and this pattern was more pronounced in mortality for circulatory and respiratory diseases. The excess mortality fractions and YLL attributable to PM_2.5_ were also generally the highest in individuals aged 0–59 years, compared to other age groups.

Our findings are generally consistent with previous large data studies. A large multi‐country time‐series study including 652 cities revealed that the two‐day moving average of PM_2.5_ was associated with increases in daily all‐cause, cardiovascular, and respiratory mortalities (Liu et al., 2019). Another large Medicare case‐crossover study (primarily consisting of individuals 65 years or older) also reported that a short‐term increase of 10 μg/m^3^ in PM_2.5_ was associated with a relative increase of 1.05% in daily mortality rate (Di, Dai, et al., 2017). Additionally, a cross‐sectional study in four countries (China, Italy, the US, and Germany) with around 9 million deaths found that a 10 μg/m^3^ increase in daily PM_2.5_ concentration was related to an increase in daily mortality per 100,000 people ranging from 0.01 to 0.10 (Ma et al., 2024).

The major finding of this study is that the risk estimates of PM_2.5_ on mortality, especially mortality for circulatory or respiratory diseases, were higher in people aged 0–59 or 60–69 years than in other older age groups, based on the point estimates. In addition to them, through more specific mortality causes analysis, we conjecture that cerebrovascular disease and pneumonia were closely related to the higher PM_2.5_‐mortality risk in the younger populations. A recent study in the United States reported a similar pattern that the all‐cause mortality risk due to PM_2.5_ was generally higher in individuals aged 45–65 years than those aged 65–75 years (Liu et al., 2022). Another Medicare study revealed that the excess relative risks of hospitalization via the emergency room for cardiovascular and respiratory diseases were higher in individuals aged less than 49 years compared to other older ages (Sun et al., 2024). Our results suggest that the younger populations, who have not been addressed as a high‐risk group regarding PM_2.5_‐mortality risk, should be recognized as new high‐risk populations, particularly if they have underlying health conditions associated with severe cardiovascular or respiratory diseases.

Several plausible hypotheses can partly explain the high PM_2.5_ risks in the younger populations. First, we conjecture that occupational (i.e., economic activities) and living factors might be associated. Previous studies showing the higher environmental exposure risks in younger populations suggested the hypothesis that younger adults may be more likely to engage in outdoor and occupational activities than older adults (Lee et al., 2021; Samoli et al., 2009).

Second, in South Korea, younger populations have poorer health behaviors than older populations. According to the Korean National Health and Nutrition Examination Survey in 2018 (Kim et al., 2018), the current smoking % was higher in middle‐aged groups (26.7% and 22.8% in 40–49 years and 50–59 years) than in older age groups (6.6% in 70 years or older). The monthly drinking %s (the % of adults who drank more than two times a week on average in the past year: 7 cups for males and 5 cups for females) were 62.8% (40–49 years) and 56.2% (50–59 years), which were higher than those of people aged 70 years or older (31.4%). It has been widely identified that smoking and alcohol consumption and their interactions increase the risk of mortality, especially cardiovascular deaths (Thadhani et al., 2002). These behaviors also increase the risk of respiratory mortality via increases in oxidative stress and inflammations that can cause or degenerate pulmonary diseases (Anderson & Ferris, 1962). Especially, regarding smoking, previous epidemiological studies have reported that there are potential synergisms between smoking behavior and mortality outcomes (Turner et al., 2014, 2017). Therefore, we cautiously conjecture that these higher outdoor activities and poor health behaviors among younger individuals could be plausible factors contributing to the higher PM_2.5_‐related mortality risks.

In addition, although the related statistical evidence was considerably weak, we found that accessibility to emergency medical facilities in the living sphere was associated with a lower association between PM_2.5_ and non‐accidental mortalities. Previous studies reported that exposure to green spaces might decrease the risk of death by improving physical activities and reducing air pollution and heat exposure (Fong et al., 2018; Lee et al., 2023). In addition, high accessibility to emergency medical facilities has been suggested as a crucial factor in reducing mortality (Azimi et al., 2021; Rea et al., 2003). Therefore, these findings could be considered for establishing community‐level action plans to reduce the risks of PM_2.5_ on health. However, we have to acknowledge the point that the relationship between regional accessibility to facilities and PM_2.5_‐mortality risk was not statistically strong, and we could not examine the potential explanations for the weak association. Thus, these results should be interpreted carefully, especially from the perspectives of statistical reliability and reproducibility.

This study has several strengths. First, to our knowledge, this is the first and largest nationwide study evaluating the association between short‐term exposure to PM_2.5_ and mortality in South Korea with various high‐risk populations. This indicates that our PM_2.5_ risk estimates may reduce biases compared to previous studies performed in selected metropolitan areas (Kim et al., 2020). Second, by examining diverse sub‐populations, our study suggests the necessity for establishing new action plans to mitigate the PM_2.5_ risks to health, especially for younger populations with specific medical conditions. Third, we provide marginal evidence that improving accessibility to parks in the living space and emergency medical facilities might be related to reducing PM_2.5_ risks of mortalities. Lastly, this study investigated the benefits of stricter PM_2.5_ mitigation policies by evaluating the mortality burden due to non‐compliance with WHO guidelines. Furthermore, given the Korean air quality guideline (daily average PM_2.5_: 35 μg/m^3^), our results suggest that the government should implement stricter air pollution standards.

Nevertheless, several limitations should be acknowledged. First, although we used a time‐stratified case‐crossover design to control for confounders that did not markedly change within a month, unmeasured confounders, such as daily high‐risk activities, smoking, and drinking, can exist. Therefore, the results of this study should be interpreted carefully, especially in relation to generalizability and causality. In other words, our study estimates did not indicate a generalizable and causal association between PM_2.5_ and deaths. Second, we used district‐average PM_2.5_ concentrations as a proxy of individual‐level PM_2.5_ exposures because the national mortality data provided only district‐level addresses (the median size of districts in South Korea is 397 km^2^, which is approximately 1.7 times larger than the median size of the US zip code areas). Thus, the potential misclassification errors should be considered. Therefore, it would be beneficial if future studies could address these limitations with larger data sets with higher spatiotemporal resolutions.

## 5 Conclusion

In summary, we evaluated an association between short‐term ambient PM_2.5_ and the risk of mortality in South Korea and suggested (probably) high‐risk age groups, particularly people aged less than 60 or 70 years who have risk factors for cardiovascular and respiratory deaths. Our findings on high‐risk populations provide evidence for establishing more target‐specific strategies. Furthermore, our results also suggest the importance of implementing stricter air quality standards to mitigate the adverse health impacts of PM_2.5_.

## Conflict of Interest

The authors declare no conflicts of interest relevant to this study.

## Supporting information

Supporting Information S1

## Data Availability

Air pollution data supporting this research are available in the AiMS‐CREATE team (https://www.datascience4health.com/) with an agreement and are not accessible to the public or research community. Researchers who would like to use this data should contact with AiMS‐CREATE team via their website (https://www.datascience4health.com/) or the corresponding author, and the data can be shared under the agreement between the researchers and the AiMS‐CREATE team. Mortality data in this study are publicly available via the Statistics Korea webpage (https://mdis.kostat.go.kr/dwnlSvc/ofrSurvSearch.do?curMenuNo=UI_POR_P9240). The webpage is only available in Korean. English readers can receive the guidance from the webpage in English (https://mdis.kostat.go.kr/eng/pageLink.do?link=mdisService).

## References

[gh270055-bib-0001] Anderson, D. O. , & Ferris, B. G. (1962). Role of tobacco smoking in the causation of chronic respiratory disease. New England Journal of Medicine, 267(16), 787–794. 10.1056/NEJM196210182671601 14012834

[gh270055-bib-0002] Azimi, A. , Bagheri, N. , Mostafavi, S. M. , Furst, M. A. , Hashtarkhani, S. , Amin, F. H. , et al. (2021). Spatial‐time analysis of cardiovascular emergency medical requests: Enlightening policy and practice. BMC Public Health, 21(1), 7. 10.1186/s12889-020-10064-1 33397340 PMC7780406

[gh270055-bib-0003] Bell, M. L. , Dominici, F. , & Samet, J. M. (2005). A meta‐analysis of time‐series studies of ozone and mortality with comparison to the national morbidity, mortality, and air pollution study. Epidemiology, 16(4), 436–445. 10.1097/01.ede.0000165817.40152.85 15951661 PMC3581312

[gh270055-bib-0004] Byun, G. , Kim, S. , Choi, Y. , Kim, A. , Team, A.‐C. , Lee, J.‐T. , & Bell, M. L. (2024). Long‐term exposure to PM_2.5_ and mortality in a national cohort in South Korea: Effect modification by community deprivation, medical infrastructure, and greenness. BMC Public Health, 24(1), 1266. 10.1186/s12889-024-18752-y 38720292 PMC11080206

[gh270055-bib-0005] Di, Q. , Dai, L. , Wang, Y. , Zanobetti, A. , Choirat, C. , Schwartz, J. D. , & Dominici, F. (2017). Association of short‐term exposure to air pollution with mortality in older adults. Journal of the American Medical Association, 318(24), 2446–2456. 10.1001/jama.2017.17923 29279932 PMC5783186

[gh270055-bib-0006] Di, Q. , Wang, Y. , Zanobetti, A. , Wang, Y. , Koutrakis, P. , Choirat, C. , et al. (2017). Air pollution and mortality in the medicare population. New England Journal of Medicine, 376(26), 2513–2522. 10.1056/NEJMoa1702747 28657878 PMC5766848

[gh270055-bib-0007] Fong, K. C. , Hart, J. E. , & James, P. (2018). A review of epidemiologic studies on greenness and health: Updated literature through 2017. Current Environmental Health Reports, 5(1), 77–87. 10.1007/s40572-018-0179-y 29392643 PMC5878143

[gh270055-bib-0008] Kim, D. , Jeong, J. , Yunsil, K. , Yunhyung, K. , & Kim, Y. T. (2018). The construction of database of community health outcomes and health determinants in the Republic of Korea. Public Health Wkly Rep KCDC, (11), 979–983.

[gh270055-bib-0009] Kim, Y. , Oh, J. , Kim, S. , Kim, A. , Park, J. , Ahn, S. , et al. (2024). Relationship between short‐term ozone exposure, cause‐specific mortality, and high‐risk populations: A nationwide, time‐stratified, case‐crossover study. Environmental Research, 261, 119712. 10.1016/j.envres.2024.119712 39096989

[gh270055-bib-0010] Kim, Y.‐o. , Lee, W. , Kim, H. , & Cho, Y. (2020). Social isolation and vulnerability to heatwave‐related mortality in the urban elderly population: A time‐series multi‐community study in Korea. Environment International, 142, 105868. 10.1016/j.envint.2020.105868 32593050

[gh270055-bib-0011] Lee, W. , Ebi, K. L. , Kim, Y. , Hashizume, M. , Honda, Y. , Hideki, H. , et al. (2021). Heat‐mortality risk and the population concentration of metropolitan areas in Japan: A nationwide time‐series study. International Journal of Epidemiology, 50(2), 602–612. 10.1093/ije/dyaa245 33346831

[gh270055-bib-0012] Lee, W. , Heo, S. , Stewart, R. , Wu, X. , Fong, K. C. , Son, J.‐Y. , et al. (2023). Associations between greenness and kidney disease in Massachusetts: The US Medicare longitudinal cohort study. Environment International, 173, 107844. 10.1016/j.envint.2023.107844 36841189

[gh270055-bib-0013] Lee, W. , Prifti, K. , Kim, H. , Kim, E. , Yang, J. , Min, J. , et al. (2022). Short‐term exposure to air pollution and attributable risk of kidney diseases: A nationwide time‐series study. Epidemiology, 33(1), 17–24. 10.1097/ede.0000000000001430 34711735

[gh270055-bib-0014] Lipfert, F. W. (2017). A critical review of the ESCAPE project for estimating long‐term health effects of air pollution. Environment International, 99, 87–96. 10.1016/j.envint.2016.11.028 27939950

[gh270055-bib-0015] Liu, C. , Chen, R. , Sera, F. , Vicedo‐Cabrera Ana, M. , Guo, Y. , Tong, S. , et al. (2019). Ambient particulate air pollution and daily mortality in 652 cities. New England Journal of Medicine, 381(8), 705–715. 10.1056/NEJMoa1817364 31433918 PMC7891185

[gh270055-bib-0016] Liu, R. A. , Wei, Y. , Qiu, X. , Kosheleva, A. , & Schwartz, J. D. (2022). Short term exposure to air pollution and mortality in the US: A double negative control analysis. Environmental Health, 21(1), 81. 10.1186/s12940-022-00886-4 36068579 PMC9446691

[gh270055-bib-0017] Ma, Y. , Nobile, F. , Marb, A. , Dubrow, R. , Stafoggia, M. , Breitner, S. , et al. (2024). Short‐term exposure to fine particulate matter and nitrogen dioxide and mortality in 4 countries. Journal of the American Medical Association Network Open, 7(3), e2354607. 10.1001/jamanetworkopen.2023.54607 PMC10907920 38427355

[gh270055-bib-0018] Ma, Y. , Zang, E. , Opara, I. , Lu, Y. , Krumholz, H. M. , & Chen, K. (2023). Racial/ethnic disparities in PM_2.5_‐attributable cardiovascular mortality burden in the United States. Nature Human Behaviour, 7(12), 2074–2083. 10.1038/s41562-023-01694-7 PMC10901568 37653149

[gh270055-bib-0019] Min, J. , Lee, W. , Kang, D.‐H. , Ahn, S. , Kim, A. , Kang, C. , et al. (2024). Air pollution and acute kidney injury with comorbid disease: A nationwide case‐crossover study in South Korea. Environmental Research, 260, 119608. 10.1016/j.envres.2024.119608 39002627

[gh270055-bib-0020] Park, J. , Kim, A. , Kim, Y. , Choi, M. , Yoon, T. H. , Kang, C. , et al. (2024). Association between heat and hospital admissions in people with disabilities in South Korea: A nationwide, case‐crossover study. The Lancet Planetary Health, 8(4), e217–e224. 10.1016/S2542-5196(24)00027-5 38580423

[gh270055-bib-0021] Rea, T. D. , Eisenberg, M. S. , Becker, L. J. , Lima, A. R. , Fahrenbruch, C. E. , Copass, M. K. , & Cobb, L. A. (2003). Emergency medical services and mortality from heart disease: A community study. Annals of Emergency Medicine, 41(4), 494–499. 10.1067/mem.2003.149 12658249

[gh270055-bib-0022] Samoli, E. , Zanobetti, A. , Schwartz, J. , Atkinson, R. , LeTertre, A. , Schindler, C. , et al. (2009). The temporal pattern of mortality responses to ambient ozone in the APHEA project. Journal of Epidemiology & Community Health, 63(12), 960–966. 10.1136/jech.2008.084012 19648130

[gh270055-bib-0023] Shaddick, G. , Thomas, M. L. , Mudu, P. , Ruggeri, G. , & Gumy, S. (2020). Half the world’s population are exposed to increasing air pollution. npj Climate and Atmospheric Science, 3(1), 23. 10.1038/s41612-020-0124-2

[gh270055-bib-0024] Sun, S. , Weinberger, K. R. , Nori‐Sarma, A. , Spangler, K. R. , Sun, Y. , Dominici, F. , & Wellenius, G. A. (2021). Ambient heat and risks of emergency department visits among adults in the United States: Time stratified case crossover study. BMJ, 375, e065653. 10.1136/bmj-2021-065653 34819309 PMC9397126

[gh270055-bib-0025] Sun, Y. , Milando, C. W. , Spangler, K. R. , Wei, Y. , Schwartz, J. , Dominici, F. , et al. (2024). Short term exposure to low level ambient fine particulate matter and natural cause, cardiovascular, and respiratory morbidity among US adults with health insurance: Case time series study. BMJ, 384, e076322. 10.1136/bmj-2023-076322 38383039 PMC10879982

[gh270055-bib-0026] Thadhani, R. , Camargo, C. A., Jr. , Stampfer, M. J. , Curhan, G. C. , Willett, W. C. , & Rimm, E. B. (2002). Prospective study of moderate alcohol consumption and risk of hypertension in young women. Archives of Internal Medicine, 162(5), 569–574. 10.1001/archinte.162.5.569 11871925

[gh270055-bib-0027] Turner, M. C. , Cohen, A. , Burnett, R. T. , Jerrett, M. , Diver, W. R. , Gapstur, S. M. , et al. (2017). Interactions between cigarette smoking and ambient PM_2.5_ for cardiovascular mortality. Environmental Research, 154, 304–310. 10.1016/j.envres.2017.01.024 28142053

[gh270055-bib-0028] Turner, M. C. , Cohen, A. , Jerrett, M. , Gapstur, S. M. , Diver, W. R. , Pope, C. A., III. , et al. (2014). Interactions between cigarette smoking and fine particulate matter in the risk of lung cancer mortality in cancer prevention study II. American Journal of Epidemiology, 180(12), 1145–1149. 10.1093/aje/kwu275 25395026

[gh270055-bib-0029] Wei, Y. , Wang, Y. , Di, Q. , Choirat, C. , Wang, Y. , Koutrakis, P. , et al. (2019). Short term exposure to fine particulate matter and hospital admission risks and costs in the Medicare population: Time stratified, case crossover study. BMJ, 367, l6258. 10.1136/bmj.l6258 31776122 PMC6880251

[gh270055-bib-0030] Wei, Y. , Wang, Y. , Wu, X. , Di, Q. , Shi, L. , Koutrakis, P. , et al. (2020). Causal effects of air pollution on mortality rate in Massachusetts. American Journal of Epidemiology, 189(11), 1316–1323. 10.1093/aje/kwaa098 32558888 PMC7604530

[gh270055-bib-0031] Wu, X. , Braun, D. , Schwartz, J. , Kioumourtzoglou, M. A. , & Dominici, F. (2020). Evaluating the impact of long‐term exposure to fine particulate matter on mortality among the elderly. Science Advances, 6(29), eaba5692. 10.1126/sciadv.aba5692 32832626 PMC7439614

[gh270055-bib-0032] Di, Q. , Amini, H. , Shi, L. , Kloog, I. , Silvern, R. , Kelly, J. , et al. (2019). An ensemble‐based model of PM_2.5_ concentration across the contiguous United States with high spatiotemporal resolution. Environment International, 130, 104909. 10.1016/j.envint.2019.104909 31272018 PMC7063579

[gh270055-bib-0033] Gasparrini, A. , Guo, Y. , Hashizume, M. , Lavigne, E. , Zanobetti, A. , Schwartz, J. , et al. (2015). Mortality risk attributable to high and low ambient temperature: A multicountry observational study. The Lancet, 386(9991), 369–375. 10.1016/S0140-6736(14)62114-0 PMC4521077 26003380

[gh270055-bib-0034] Kim, H. , Jang, H. , Lee, W. , Oh, J. , Lee, J.‐Y. , Kim, M.‐H. , et al. (2024). Association between long‐term PM_2.5_ exposure and risk of Kawasaki disease in children: A nationwide longitudinal cohort study. Environmental Research, 244, 117823. 10.1016/j.envres.2023.117823 38072109

[gh270055-bib-0035] Lee, W. , Choi, M. , Bell, M. L. , Kang, C. , Jang, J. , Song, I. , et al. (2021). Effects of urbanization on vulnerability to heat‐related mortality in urban and rural areas in South Korea: A nationwide district‐level time‐series study. International Journal of Epidemiology, 51(1), 111–121. 10.1093/ije/dyab148 34386817

[gh270055-bib-0036] Park, J. , Kang, C. , Min, J. , Kim, E. , Song, I. , Jang, H. , et al. (2023). Association of long‐term exposure to air pollution with chronic sleep deprivation in South Korea: A community‐level longitudinal study, 2008–2018. Environmental Research, 228, 115812. 10.1016/j.envres.2023.115812 37030407

[gh270055-bib-0037] Xu, S. , Marcon, A. , Bertelsen, R. J. , Benediktsdottir, B. , Brandt, J. , Engemann, K. , et al. (2023). Long‐term exposure to low‐level air pollution and greenness and mortality in Northern Europe. The Life‐GAP project. Environment International, 181, 108257. 10.1016/j.envint.2023.108257 37857189

