# Supplementary material for: Short‐Term Exposure to Fine Particulate Matter (PM2.5), Cause Specific‐Mortality, and High‐Risk Populations: A Nationwide Time‐Stratified Case‐Crossover Study
Source: Geohealth. 2025 Sep 26;9(10):e2024GH001214. doi: 10.1029/2024GH001214 (PMC12474854; doi:10.1029/2024GH001214)
Supplement: Supplementary file 1 — Supporting Information S1 [file GH2-9-e2024GH001214-s001.docx]

**Supporting Information**

**Title:** **Short-term exposure to fine particulate matter (PM_2.5_), cause specific-mortality, and high-risk populations: a nationwide time-stratified case-crossover study**

Seoyeong Ahn^1,2^, Jieun Oh^3^, Hyewon Yun^4^, Harin Min^4^, Yejin Kim^1^, Cinoo Kang^3^, Sojin An^4^, Ayoung Kim^3^, Dohoon Kwon^3^, Jinah Park^3^, Whanhee Lee^2,5¥^

**Affiliations**

1 Department of Information Convergence Engineering, College of Information and Biomedical Engineering, Pusan National University, Yangsan, South Korea

2 Environmental Health Center for Climate Change, Pusan National University, Yangsan, South Korea

3 Department of Public Health Sciences, Graduate School of Public health, Seoul National University, Seoul, South Korea

4 Graduate School of Data Science, Pusan National University, Busan, South Korea

5 School of Biomedical Convergence Engineering, College of Information and Biomedical Engineering, Pusan National University, Yangsan, South Korea

**Corresponding Author: Whanhee Lee**

School of Biomedical Convergence Engineering, College of Information and Biomedical Engineering, Pusan National University, 49 Busandaehak-ro, Mulgeum-eup, Yangsan-si, Gyeongsangnam-do 50612, South Korea. Telephone: (82) 51-510-8599. E-mail: [whanhee.lee@pusan.ac.kr](mailto:whanhee.lee@pusan.ac.kr)

**Table of Contents**

**1. Causes of death - non-accidental causes of death**

**2. Air Pollution Prediction Model**

**3. District-level indicators**

**4. The excess mortality and Years of life lost from mortality attributable to PM_2.5_**

**5. Supplementary Tables**

- Table S1. Summary statistics on the performance of the PM_2.5_ and NO_2_ prediction models during the study period (2015 to 2019)
- Table S2. The association between short-term exposure PM_2.5_ (lag 0-1) and mortality by specific causes of death
- Table S3. Effect modification by district-level indicators in the PM_2.5_-mortality risk with specific causes of death
- Table S4. Excess deaths attributable to ambient short-term PM_2.5_ exposure (lag 0-1) for all-cause mortality
- Table S5. Sensitivity Analysis

**6. Supplementary Figures**

- Figure S1. The association between short-term exposure PM_2.5_ (lag 0-1) and mortality by age group and sex.
- Figure S2. Effect modification by district-level indicators in the PM_2.5_-mortality risk for each sex

**1. Causes of death - non-accidental causes of death**

We used the International Classification of Disease 10th Revision (ICD-10) to define causes of death. Non-accidental deaths included following causes: certain infectious and parasitic diseases (A00-B99), neoplasms (C00-D48), diseases of the blood and blood-forming organs and certain disorders involving the immune mechanism (D50-D89), endocrine, nutritional and metabolic diseases (E00-E90), mental and behavioral disorders (F00-F99), diseases of the nervous system (G00-G99), diseases of the eye and adnexa (H00-H59), diseases of the ear and mastoid process (H60-H95), diseases of the circulatory system (I00-I99), diseases of the respiratory system (J00-J99), diseases of the digestive system (K00-K93), diseases of the skin and subcutaneous tissue (L00-L99), diseases of the musculoskeletal system and connective tissue (M00-M99), diseases of the genitourinary system (N00-N99), pregnancy, childbirth and the puerperium (O00-O99), certain conditions originating in the perinatal period (P00-96), congenital malformations, deformations and chromosomal abnormalities( Q00-99) and symptoms, signs and abnormal clinical and laboratory findings, not elsewhere classified (R00-99).

**2. Air Pollution Prediction Model**

This model was provided air pollution data by AiMS-CREATE team (hereafter “the team”), which is a research network for environmental health between Pusan National University and Seoul National University in Korea, and their products were used in previously published studies.(Kim et al., 2024; Park et al., 2023)

**(1) Information on monitoring station**

As response variables for the air pollution prediction modeling (i.e. as true values), we collected ground-level hourly measured PM_2.5_ concentrations from the Air Korea database provided by the Ministry of Environment (URL: <https://www.airkorea.or.kr/>) Korea from Jan. 01, 2015 to Dec 31, 2022. To reduce potential biases, we used concentration data from monitoring sites with observations for ≥ 9 months per year (observed 75% or over). The total number of selected monitoring sites was 483 for PM_2.5_ and NO_2_ (105 stations in 2015 to 483 stations in 2022). From the selected monitoring sites, we calculated the average ambient concentrations of daily PM_2.5_ and NO_2_.

**(2) Explanatory variables for prediction models with single machine learning algorithms**

**(2.1) Satellite-derived data**

Daily variables based on satellite remote sensing from 2015 through 2022 were used as major predictors. First, the team used 1km^2^ grid cell shape file provided by Statistics Korea. Then, the team collected a total of 47 daily remote sensing variables through the Google Earth Engine (URL: <https://earthengine.google.com/>), and these variables included daily aerosol optical depth, meteorological, surface reflectance and landcover data from different satellite-based databases (the Table below). If the spatial resolution of certain variables was higher than 1km^2^, then we calculated and allocated the average values for each variable inside the boundary of each 1 km^2^ grid cell. Also, if the spatial resolution of certain variables was lower than 1 km^2^, we allocated the nearest values to the centroid of the 1 km^2^ grid cells and calculated the average values when two or more values were included in each 1 km^2^ grid cell Then, we used the *missForest* method to impute missing values in 1 km^2^ grid cell which were not measured with each satellite. Finally, we standardized the satellite-driven data to make zero-mean and unit-variance.

| **Data source** | **Predictor variables** | **Spatiotemporal**  **Resolution** |
| --- | --- | --- |
| ERA5-Land Daily Aggregated - ECMWF Climate Reanalysis | Temperature_2m | 11.13 km, Hourly |
|  | Skin_temperature |  |
|  | Soil_temperature_level_1 |  |
|  | Leaf_area_index_low_vegetation |  |
|  | Leaf_area_index_high_vegetation |  |
|  | Total_precipitation |  |
|  | Surface_pressure |  |
|  | u_component_of_wind_10m |  |
|  | v_component_of_wind_10m |  |
| MOD09GA.061 Terra Surface Reflectance Daily Global 1km and 500m | Sur_refl_b01 | 500 m, Daily |
|  | Sur_refl_b02 |  |
|  | Sur_refl_b03 |  |
|  | Sur_refl_b07 |  |
| MOD11A1.061 Terra Land Surface Temperature and Emissivity Daily Global 1km | Emis_31 | 1 km, Daily |
|  | Emis_32 |  |
|  | LST_Night_1km |  |
|  | LST_Day_1km |  |
| CFSV2: NCEP Climate Forecast System Version 2, 6-Hourly Products | Maximum_specific_humidity_at_2m_height_above_ground_6_hour_interval | 22.26 km, 6-hour |
|  | Minimum_specific_humidity_at_2m_height_above_ground_6_hour_interval |  |
|  | Specific_humidity_height_above_ground |  |
|  | Maximum_temperature_height_above_ground_6_hour_interval |  |
|  | Minimum_temperature_height_above_ground_6_hour_interval |  |
|  | Geopotential_height_surface |  |
| MCD19A2.061: Terra & Aqua MAIAC Land Aerosol Optical Depth Daily 1km | Optical_Depth_047 | 1 km, Daily |
|  | Optical_Depth_055 |  |

**Meteorological variables:** Air pollution can be affected spatially and temporally by meteorological factors such as temperature, wind speed and direction, precipitation, humidity, and cloud droplets, the team collected satellite datasets from the 5^th^ generation European Center for Medium-Range Weather Forecasts atmospheric reanalysis (ERA5)^3^, which is archive at the Google Earth Engine. The ERA5 data (~9 to 11km^2^) includes average air temperature at 2m height (daily average), skin temperature (daily average), soil temperature in layer 1 (daily average), total precipitation (daily sums), surface pressure (daily average), 10m u-component of wind (daily average), and 10m v-component of wind (daily average).

Additionally, from the National Centers for Environmental Prediction (NCEP) Climate Forecast System (CFS), we collected maximum/minimum/mean specific humidity at 2m height (6-hour average), maximum/minimum temperature at 2m height (6-hour average) and geopotential height at surface (6-hour average). Also, from the Terra Moderate Resolution Imaging Spectroradiometer (MODIS), we used daytime/nighttime land surface temperature (daily average), emissivity with band 31 and 32 (daily average) and surface reflectance with band 1,2,3 and 7 (daily average).

**Aerosol variables:** The team used two aerosol optical depth variables: Aerosol optical depth over land retrieved in the MODIS Green band (0.55 μm) and aerosol optical depth over land retrieved in the MODIS blue band (0.47 μm). Aerosol optical depth is not received at high altitude (greater than 4.2 km) except when smoke or dust is detected; rather, this value reports a static value of 0.02 used for atmospheric correction.

**Vegetation variables:** The variables related to the vegetation in each grid were collected in the following two datasets in Google Earth Engine. With ERA5 dataset, we collected one-half of the total green leaf area per unit horizontal ground surface area for high vegetation type (daily average) and one-half of the total green leaf area per unit horizontal ground surface area for low vegetation type (daily average). Also, with MODIS dataset, we used the 16-day averaged Enhanced Vegetation Index (EVI), the second vegetation layer that can mitigate canopy background variations, preserve sensitivity in dense vegetation, and utilize the blue band to eliminate residual atmosphere contamination from smoke and sub-pixel thin cloud clouds.

**(2.2) Regional data**

**Population density:** We used population density data from the community health-related factor database sourced from the Korea Centers for Disease Control and Prevention, which comprises data pertaining to health determinants encompassing physical, environmental, and demographic factors influencing community health levels and disparities. Therefore, as population density data was collected at a Korean district-level (‘Si-gun-gu’) and its spatial resolution is bigger than 1km^2^, the same value was assigned to the grids which were included in each district.

**(3) Machine learning modeling and model ensemble**

Three machine learning-based models, namely random forest, light gradient boosting, and deep neural network, were used to predict daily PM_2.5_ and NO_2_ averages (individually) with a 1 km^2^ grid during 2015–2022. The team trained algorithm individually on all input variables (daily satellite variables with EVI and population density variables) and parameters of each algorithm selected by cross-validated grid search. To avoid overfitting, we validated each model with a cross-validation. In addition, because the number of monitoring stations in Korea has increased every year during the study period, we did a year-stratified cross-validation: training with 80% of the data and testing a prediction performance at the remaining 20% of data by each study year (from 2015 to 2022). From these procedures, we found the optimized model for each algorithm and calculated 1km^2^ daily predicted PM_2.5_ and NO_2_ concentrations.

Furthermore, in order to increase the prediction performance, we performed an ensemble approach using the generalized additive model (GAM) to incorporate these three algorithms.(Di et al., 2019) This GAM model allows to address a flexible regression for the monitoring concentrations against the estimates from each machine learning algorithm, and annual land-use variables by thin plate splines. In here, we considered annual land-use variables collected from MODIS through the Google Earth Engine (the Table below)

| **Data source** | **Predictor variables** | **Collection Period** | **Spatiotemporal**  **Resolution** |
| --- | --- | --- | --- |
| Copernicus Global Land Cover Layers: CGLS-LC100 Collection 3 | Bare-coverfraction | 2015~2019 | 100m |
|  | Crops-coverfraction |  |  |
|  | Grass-coverfraction |  |  |
|  | Shrub-coverfraction |  |  |
|  | Tree-coverfraction |  |  |
|  | Urban-coverfraction |  |  |
|  | Water-permanent-coverfraction |  |  |
|  | Water-seasonal-coverfraction |  |  |
|  | Forest_type |  |  |
| GlobCover: Global Land Cover Map | Landcover | 2009~2010 | 300m |
| MCD12Q1.061 MODIS Land Cover Type Yearly Global 500m | LC_Type1 | 2002~2021 | 500m, Yearly |
|  | LC_Type2 |  |  |
|  | LC_Type3 |  |  |
|  | LC_Type4 |  |  |
|  | LC_Type5 |  |  |
|  | LC_Prop1 |  |  |
|  | LC_Prop2 |  |  |
|  | LC_Prop3 |  |  |
|  | LC_Prop1_Assessment |  |  |
|  | LC_Prop2_Assessment |  |  |
|  | LC_Prop3_Assessment |  |  |
| MOD13A2.061 Terra Vegetation Indices 16-Day Global 1km | EVI | 2002~2020 | 1km, 16-day |

**Land-use variables:** To consider regional heterogeneity, we collected several land-use variables. From the Copernicus Global Land Service (CGLS), we used forest type with tree percentage vegetation cover bigger than 1% and percent vegetation cover for the various land cover classes including bare-sparse-vegetation, cropland, herbaceous vegetation, shrubland, forest, build-up, permanent water and seasonal water. Also, with the global land cover map based on ENVISAT's Medium Resolution Imaging Spectrometer (MERIS) Level 1B data, we applied the land cover map data.

Furthermore, there are the yearly land-use data provided by MODIS dataset. The data contains annual International Geosphere-Biosphere Programme (IGBP) classification, annual University of Maryland (UMD) classification, annual Leaf Area Index (LAI) classification, annual BIOME-Biogeochemical Cycles (BGC) classification, annual Plant Functional Types classification, LCCS1 land cover layer (confidence), LCCS1 land cover layer (confidence) and LCCS3 surface hydrology layer (confidence). As the data provides yearly data, we assigned the same value to the daily values which were included in each year.

**(4) Performance of the GAM Ensemble Prediction Model**

Among the three pollutant models with single algorithm and the GAM ensemble model, the GAM ensemble model showed the best prediction accuracy for both PM_2.5_ and NO_2_. Thus, we summarized the performance of the GAM ensemble model below:

**3. District-level indicators**

To examine the spatial difference in PM_2.5_-mortality risk according to regional characteristics, we collected data on four district-level indicators covering urbanicity, vegetation, and levels of medical accessibility. In the statistical analysis, all indicators were recalculated as average values during the entire study period, although they were provided by year.

First, to consider the risk difference by urbanicity, we collected population density (persons per km^2^), which has been addressed as one of the most suitable indicators that can reflect the urbanicity level in South Korea.(Lee et al., 2021)

Second, to address the vegetation level which has been investigated as a mitigation factor of the PM_2.5_-health risk,(Byun et al., 2024; Xu et al., 2023) we collected the accessibility to parks in the living sphere by a 1 km^2^ grid (i.e. distance to neighboring parks from each grid) from the National Geographic Information Platform (<https://map.ngii.go.kr/mn/mainPage.do>) operated by the National Geographic Information Institute, Ministry of Land, Infrastructure and Transport of South Korea. According to Korean law (Urban Parks and Green Spaces Acts), the parks in the living sphere refer to small park, children’s park, or neighboring park in urbanized areas. We aggregated the data to the district unit by averaging the park accessibility values at grid cells with centroid points inside the boundaries of each district.

Third, we obtained two variables related to medical accessibility: the number of beds in hospitals per 1,000 persons (hereafter, the number of hospital beds) and the accessibility to emergency medical facilities. We collected the district-level number of hospital beds from the community health-related factor database provided by the Korea Disease Control and Prevention Agency (Kim et al., 2018).

Finally, the accessibility to emergency medical facilities variable was collected from the National Geographic Information Platform, like the accessibility to parks in the living sphere. The variable was evaluated as the number of people living within 5 km of emergency medical facilities and was provided at a 1 km^2^ grid cell resolution across all territories of South Korea, then we averaged the values of grid cells with centroid points inside the boundary of each district.

**4. The excess mortality and Years of life lost from mortality attributable to PM_2.5_**

Risk estimates for PM_2.5_ (ORs) were translated into excess mortality and YLL (years of life lost from mortality) attributable to PM_2.5_, to demonstrate the change in mortality burden due to PM_2.5_ exposures.

First, we assigned the YLL for each death case based on the national life expectancy estimation in 2019 provided by Statistics Korea (https://kosis.kr/statHtml/statHtml.do?orgId=101&tblId =DT_1B41&conn_path=I2). We used national age-specific life expectancy (categorized by 10-year intervals) and calculated individual YLL as the difference between the age at death and life expectancy for the corresponding age categories at death. Here are the life expectancy estimates based on 10-year-old categories: 0–9 y: 83.3/ 10–19 y: 73.6/ 20–29 y: 63.7 / 30–39 y: 53.9 / 40–49 y: 44.2, 50–59 y: 34.8 / 60–69 y: 25.7 / 70–79 y: 17.1 / 80 y or older: 9.7 years.

Then, we created multiple time-series data including daily mean PM_2.5_, daily mortality counts, and daily sum of YLL for each cause of death. For each time-series data, we calculated the daily excess death and YLL attributable to PM_2.5_ for each cause of mortality using the corresponding OR (calculated from conditional logistic models for each cause in the total population) associated with the level of PM_2.5_ of each day. The sum of attributable deaths and YLL represents the total excess number of deaths and YLLs attributable to PM_2.5_, and its ratio with the total number of deaths provides the total excess fraction of such deaths attributable to PM_2.5_. Because previous research reported that the adverse impacts of PM_2.5_ persist at very low levels,(Sun et al., 2024; Wei et al., 2020) we set a minimum concentration as the reference to calculate excess deaths and YLL attributable to PM_2.5_ (i.e., excess deaths and YLL due to the whole range of PM_2.5_), although we recognize that this includes natural background concentrations. To examine the mortality burden associated with compliance with the WHO 2021 guidelines, we calculated attributable deaths and YLL only for the subset of days with PM_2.5_ levels above (i.e. non-compliance) the WHO air quality guidelines (daily average PM_2.5_ 15 µg/m^3^). We used Monte Carlo simulations to calculate the confidence intervals of each estimate, using 1,000 replicates (Gasparrini et al., 2015; Lee et al., 2022).

Further, we hypothesized that the PM_2.5_ risk on mortality could be substantially heterogeneous by age, thus, we also calculated the age group-specific daily excess deaths and YLL attributable to PM_2.5_ using the age-cause-specific ORs (estimated from the main analysis) and daily death counts and YLL by age group.

**5. Supplementary Tables**

**Table S1. Summary statistics on the performance of the PM_2.5_ and NO_2_ prediction models during the study period (2015 to 2019).** RMSE: root mean squared error, MAE: mean absolute error. RMSE and MAE are in the unit of PM_2.5_ (μg/m^3^) and NO2 (ppm)

|  |  | **R^2^** | **RMSE** | **MAE** |
| --- | --- | --- | --- | --- |
| **PM_2.5_** | **Total years** | 0.944 | 3.219 | 2.187 |
|  | **2015** | 0.889 | 3.375 | 2.179 |
|  | **2016** | 0.882 | 3.352 | 2.290 |
|  | **2017** | 0.925 | 3.283 | 2.258 |
|  | **2018** | 0.957 | 3.453 | 2.148 |
|  | **2019** | 0.965 | 3.135 | 2.138 |
| **NO_2_** | **Total years** | 0.944 | 0.002 | 0.002 |
|  | **2015** | 0.889 | 0.003 | 0.002 |
|  | **2016** | 0.882 | 0.003 | 0.002 |
|  | **2017** | 0.925 | 0.002 | 0.002 |
|  | **2018** | 0.957 | 0.002 | 0.002 |
|  | **2019** | 0.965 | 0.002 | 0.001 |

**Table S2. The association between short-term exposure PM_2.5_ (lag 0-1) and mortality by specific causes of death.** OR: odds ratio for a 10μg/m^3^ increase in PM_2.5_, P-value: P-value of OR (H_0_: OR is one)

|  | **Specific causes** | **Age group** | **OR** | **P-value** |
| --- | --- | --- | --- | --- |
| **Circulatory** | **Ischemic heart diseases** | **Total** | 1.005 | 0.303 |
|  |  | **0-59 y** | 1.000 | 0.986 |
|  |  | **60-69 y** | 0.975 | 0.045 |
|  |  | **70-79 y** | 1.01 | 0.291 |
|  |  | **80 y +** | 1.013 | 0.063 |
|  | **Cerebrovascular diseases** | **Total** | 1.008 | 0.031 |
|  |  | **0-59 y** | 1.023 | 0.028 |
|  |  | **60-69 y** | 1.008 | 0.444 |
|  |  | **70-79 y** | 1.000 | 0.989 |
|  |  | **80 y+** | 1.008 | 0.113 |
|  | **Other circulatory diseases** | **Total** | 1.007 | 0.041 |
|  |  | **0-59 y** | 1.018 | 0.133 |
|  |  | **60-69 y** | 1.009 | 0.45 |
|  |  | **70-79 y** | 1.000 | 0.995 |
|  |  | **80 y+** | 1.008 | 0.076 |
| **Respiratory** | **Pneumonia** | **Total** | 1.006 | 0.103 |
|  |  | **0-59 y** | 1.045 | 0.024 |
|  |  | **60-69 y** | 1.024 | 0.138 |
|  |  | **70-79 y** | 1.009 | 0.304 |
|  |  | **80 y +** | 1.002 | 0.684 |
|  | **Chronic Lower Respiratory Disease** | **Total** | 1.008 | 0.227 |
|  |  | **0-59 y** | 0.94 | 0.171 |
|  |  | **60-69 y** | 1.023 | 0.366 |
|  |  | **70-79 y** | 0.998 | 0.862 |
|  |  | **80 y+** | 1.014 | 0.104 |
|  | **Other Respiratory diseases** | **Total** | 1.007 | 0.328 |
|  |  | **0-59 y** | 0.997 | 0.904 |
|  |  | **60-69 y** | 1.000 | 0.999 |
|  |  | **70-79 y** | 1.013 | 0.298 |
|  |  | **80 y+** | 1.006 | 0.54 |

**Table S3. Effect modification by district-level indicators in the PM_2.5_-mortality risk with specific causes of death.** OR: odds ratio for a 10μg/m^3^ increase in PM_2.5_, P-value: P-value of OR (H_0_: OR is one)

| **Specific causes** | **Indicators** | **Category** | **OR** | **P-value** |
| --- | --- | --- | --- | --- |
| **Ischemic heart diseases** | Population density | Low | 1.002 |  |
|  |  | Middle | 1.014 | 0.410 |
|  |  | High | 1.000 | 0.839 |
|  | Distance to park | Low | 0.999 |  |
|  |  | Middle | 1.019 | 0.040 |
|  |  | High | 0.990 | 0.464 |
|  | Number of Hospital beds | Low | 1.005 |  |
|  |  | Middle | 1.001 | 0.692 |
|  |  | High | 1.009 | 0.718 |
|  | Distance to emergency medical facilities | Low | 1.003 |  |
|  |  | Middle | 1.008 | 0.630 |
|  |  | High | 1.004 | 0.978 |
| **Cerebrovascular diseases** | Population density | Low | 1.016 |  |
|  |  | Middle | 1.006 | 0.390 |
|  |  | High | 1.007 | 0.403 |
|  | Distance to park | Low | 1.006 |  |
|  |  | Middle | 1.006 | 0.976 |
|  |  | High | 1.020 | 0.191 |
|  | Number of Hospital beds | Low | 1.010 |  |
|  |  | Middle | 1.008 | 0.733 |
|  |  | High | 1.004 | 0.454 |
|  | Distance to emergency medical facilities | Low | 1.007 |  |
|  |  | Middle | 1.003 | 0.528 |
|  |  | High | 1.022 | 0.149 |
| **Other circulatory diseases** | Population density | Low | 0.991 |  |
|  |  | Middle | 1.007 | 0.154 |
|  |  | High | 1.011 | 0.055 |
|  | Distance to park | Low | 1.012 |  |
|  |  | Middle | 1.008 | 0.659 |
|  |  | High | 0.986 | 0.013 |
|  | Number of Hospital beds | Low | 1.006 |  |
|  |  | Middle | 1.002 | 0.604 |
|  |  | High | 1.018 | 0.155 |
|  | Distance to emergency medical facilities | Low | 1.014 |  |
|  |  | Middle | 1.005 | 0.231 |
|  |  | High | 0.991 | 0.014 |
| **Pneumonia** | Population density | Low | 1.017 |  |
|  |  | Middle | 1.008 | 0.422 |
|  |  | High | 1.003 | 0.204 |
|  | Distance to park | Low | 1.007 |  |
|  |  | Middle | 1.004 | 0.740 |
|  |  | High | 1.012 | 0.600 |
|  | Number of Hospital beds | Low | 1.006 |  |
|  |  | Middle | 0.998 | 0.355 |
|  |  | High | 1.019 | 0.178 |
|  | Distance to emergency medical facilities | Low | 1.006 |  |
|  |  | Middle | 1.002 | 0.649 |
|  |  | High | 1.016 | 0.300 |
| **Chronic Lower Respiratory Disease** | Population density | Low | 0.988 |  |
|  |  | Middle | 1.014 | 0.173 |
|  |  | High | 1.010 | 0.225 |
|  | Distance to park | Low | 1.009 |  |
|  |  | Middle | 1.013 | 0.742 |
|  |  | High | 0.995 | 0.478 |
|  | Number of Hospital beds | Low | 1.002 |  |
|  |  | Middle | 1.025 | 0.138 |
|  |  | High | 0.994 | 0.638 |
|  | Distance to emergency medical facilities | Low | 1.008 |  |
|  |  | Middle | 1.016 | 0.582 |
|  |  | High | 0.996 | 0.490 |
| **Other Respiratory diseases** | Population density | Low | 1.005 |  |
|  |  | Middle | 1.006 | 0.930 |
|  |  | High | 1.007 | 0.903 |
|  | Distance to park | Low | 1.010 |  |
|  |  | Middle | 1.000 | 0.439 |
|  |  | High | 1.012 | 0.939 |
|  | Number of Hospital beds | Low | 1.006 |  |
|  |  | Middle | 1.004 | 0.875 |
|  |  | High | 1.012 | 0.693 |
|  | Distance to emergency medical facilities | Low | 1.012 |  |
|  |  | Middle | 0.999 | 0.351 |
|  |  | High | 1.004 | 0.656 |

**Table S4. Excess deaths attributable to ambient short-term PM_2.5_ exposure (lag 0-1) for all-cause mortality**

|  |  | **All-range of PM_2.5_** | **WHO guideline compliance** |
| --- | --- | --- | --- |
| **Cause of death** | **Age group** | **Excess deaths** | **Excess deaths** |
| **Non-accidental** | **Total** | 23878.01  (17392.67-30542.7) | 10209.48  (7429.88-13069.64) |
|  | **0-59 years** | 3706.66  (1272.44-6188.17) | 1597.41  (546.96-2671.90) |
|  | **60-69 years** | 2810.60  (458.64-5206.88) | 1197.86  (194.92-2223.35) |
|  | **70-79 years** | 3769.13  (422.08-7191.84) | 1608.26  (179.72-3072.95) |
|  | **80 y and older** | 13541.84  (9113.61-18082.36) | 5790.07  (3891.61-7740.30) |
| **Circulatory disease** | **Total** | 5106.19  (1919.65-8363.58) | 2189.51  (821.46-3591.77) |
|  | **0-59 years** | 1181.21  (146.85-2217.87) | 512.64  (63.34-966.46) |
|  | **60-69 years** | -92.60  (-1150.88-966.62) | -39.02  (-489.90-416.01) |
|  | **70-79 years** | 427.07  (-1215.99-2089.10) | 183.79  (-519.50-899.23) |
|  | **80 y and older** | 3550.59  (1233.77-5910.02) | 1519.20  (526.43-2533.95) |
| **Respiratory disease** | **Total** | 2685.48  (346.27-5067.84) | 1155.19  (148.52-2184.16) |
|  | **0-59 years** | 274.90  (-187.59-720.39) | 120.85  (-81.14-318.90) |
|  | **60-69 years** | 466.97  (-160.96-1083.07) | 201.91  (-68.77-471.20) |
|  | **70-79 years** | 709.99  (-469.7-1894.38) | 305.66  (-200.79-818.01) |
|  | **80 y and older** | 1231.66  (-647.63-3139.02) | 529.56  (-277.33-1352.17) |

**Table S5. Sensitivity Analysis**

| **Cause of death** | **Model specification** | **Age category** | **OR** | **P-value** |
| --- | --- | --- | --- | --- |
| **Non-accidental death** | **PM_2.5_ (Lag 0-2)** | Total | 1.008 | 0.000 |
|  |  | 0-59 y | 1.010 | 0.002 |
|  |  | 60-69 y | 1.007 | 0.032 |
|  |  | 70-79 y | 1.004 | 0.082 |
|  |  | 80 y+ | 1.010 | 0.000 |
|  | **PM_2.5_ (Lag 0-3)** | Total | 1.008 | 0.000 |
|  |  | 0-59 y | 1.010 | 0.002 |
|  |  | 60-69 y | 1.008 | 0.029 |
|  |  | 70-79 y | 1.003 | 0.186 |
|  |  | 80 y+ | 1.010 | 0.000 |
|  | **Temperature (df=7)** | Total | 1.008 | 0.000 |
|  |  | 0-59 y | 1.008 | 0.003 |
|  |  | 60-69 y | 1.007 | 0.021 |
|  |  | 70-79 y | 1.005 | 0.032 |
|  |  | 80 y+ | 1.009 | 0.000 |
|  | **Temperature (df=5)** | Total | 1.008 | 0.000 |
|  |  | 0-59 y | 1.009 | 0.003 |
|  |  | 60-69 y | 1.007 | 0.021 |
|  |  | 70-79 y | 1.005 | 0.027 |
|  |  | 80 y+ | 1.009 | 0.000 |
|  | **Temperature (Lag 0-1)** | Total | 1.008 | 0.000 |
|  |  | 0-59 y | 1.009 | 0.003 |
|  |  | 60-69 y | 1.008 | 0.009 |
|  |  | 70-79 y | 1.005 | 0.042 |
|  |  | 80 y+ | 1.009 | 0.000 |
|  | **Temperature (Lag 0-6)** | Total | 1.006 | 0.000 |
|  |  | 0-59 y | 1.006 | 0.024 |
|  |  | 60-69 y | 1.005 | 0.094 |
|  |  | 70-79 y | 1.003 | 0.163 |
|  |  | 80 y+ | 1.008 | 0.000 |
|  | **NO_2_ (Lag 0-1)** | Total | 1.008 | 0.000 |
|  |  | 0-59 y | 1.009 | 0.003 |
|  |  | 60-69 y | 1.008 | 0.013 |
|  |  | 70-79 y | 1.006 | 0.013 |
|  |  | 80 y+ | 1.009 | 0.000 |
| **Circulatory death** | **PM_2.5_ (Lag 0-2)** | Total | 1.007 | 0.002 |
|  |  | 0-59 y | 1.016 | 0.023 |
|  |  | 60-69 y | 0.999 | 0.864 |
|  |  | 70-79 y | 1.001 | 0.878 |
|  |  | 80 y+ | 1.010 | 0.002 |
|  | **PM_2.5_ (Lag 0-3)** | Total | 1.008 | 0.004 |
|  |  | 0-59 y | 1.013 | 0.094 |
|  |  | 60-69 y | 1.005 | 0.573 |
|  |  | 70-79 y | 1.000 | 0.949 |
|  |  | 80 y+ | 1.010 | 0.005 |
|  | **Temperature (df=7)** | Total | 1.007 | 0.002 |
|  |  | 0-59 y | 1.015 | 0.026 |
|  |  | 60-69 y | 0.999 | 0.854 |
|  |  | 70-79 y | 1.002 | 0.636 |
|  |  | 80 y+ | 1.009 | 0.003 |
|  | **Temperature (df=5)** | Total | 1.007 | 0.001 |
|  |  | 0-59 y | 1.015 | 0.025 |
|  |  | 60-69 y | 0.999 | 0.848 |
|  |  | 70-79 y | 1.002 | 0.582 |
|  |  | 80 y+ | 1.009 | 0.003 |
|  | **Temperature (Lag 0-1)** | Total | 1.007 | 0.002 |
|  |  | 0-59 y | 1.013 | 0.052 |
|  |  | 60-69 y | 1.001 | 0.870 |
|  |  | 70-79 y | 1.002 | 0.653 |
|  |  | 80 y+ | 1.009 | 0.003 |
|  | **Temperature (Lag 0-6)** | Total | 1.005 | 0.029 |
|  |  | 0-59 y | 1.012 | 0.053 |
|  |  | 60-69 y | 0.997 | 0.624 |
|  |  | 70-79 y | 0.999 | 0.846 |
|  |  | 80 y+ | 1.007 | 0.014 |
|  | **NO_2_ (Lag 0-1)** | Total | 1.007 | 0.002 |
|  |  | 0-59 y | 1.017 | 0.015 |
|  |  | 60-69 y | 0.999 | 0.921 |
|  |  | 70-79 y | 1.002 | 0.728 |
|  |  | 80 y+ | 1.010 | 0.003 |
| **Respiratory death** | **PM_2.5_ (Lag 0-2)** | Total | 1.008 | 0.023 |
|  |  | 0-59 y | 1.029 | 0.080 |
|  |  | 60-69 y | 1.013 | 0.298 |
|  |  | 70-79 y | 1.005 | 0.455 |
|  |  | 80 y+ | 1.007 | 0.109 |
|  | **PM_2.5_ (Lag 0-3)** | Total | 1.008 | 0.023 |
|  |  | 0-59 y | 1.028 | 0.128 |
|  |  | 60-69 y | 1.012 | 0.398 |
|  |  | 70-79 y | 1.003 | 0.649 |
|  |  | 80 y+ | 1.009 | 0.058 |
|  | **Temperature (df=7)** | Total | 1.007 | 0.028 |
|  |  | 0-59 y | 1.018 | 0.234 |
|  |  | 60-69 y | 1.017 | 0.143 |
|  |  | 70-79 y | 1.007 | 0.256 |
|  |  | 80 y+ | 1.005 | 0.214 |
|  | **Temperature (df=5)** | Total | 1.007 | 0.025 |
|  |  | 0-59 y | 1.018 | 0.242 |
|  |  | 60-69 y | 1.017 | 0.143 |
|  |  | 70-79 y | 1.007 | 0.228 |
|  |  | 80 y+ | 1.005 | 0.204 |
|  | **Temperature (Lag 0-1)** | Total | 1.008 | 0.013 |
|  |  | 0-59 y | 1.023 | 0.140 |
|  |  | 60-69 y | 1.017 | 0.159 |
|  |  | 70-79 y | 1.008 | 0.214 |
|  |  | 80 y+ | 1.006 | 0.130 |
|  | **Temperature (Lag 0-6)** | Total | 1.005 | 0.123 |
|  |  | 0-59 y | 1.014 | 0.352 |
|  |  | 60-69 y | 1.013 | 0.249 |
|  |  | 70-79 y | 1.004 | 0.459 |
|  |  | 80 y+ | 1.003 | 0.399 |
|  | **NO_2_ (Lag 0-1)** | Total | 1.006 | 0.077 |
|  |  | 0-59 y | 1.020 | 0.194 |
|  |  | 60-69 y | 1.013 | 0.273 |
|  |  | 70-79 y | 1.009 | 0.019 |
|  |  | 80 y+ | 1.003 | 0.494 |

**6. Supplementary Figures**

**Figure S1. The association between short-term exposure PM_2.5_ (lag 0-1) and mortality by age group and sex.** OR: odds ratio for a 10μg/m3 increase in PM_2.5_, P-value: P-value of OR (H_0_: OR is zero)


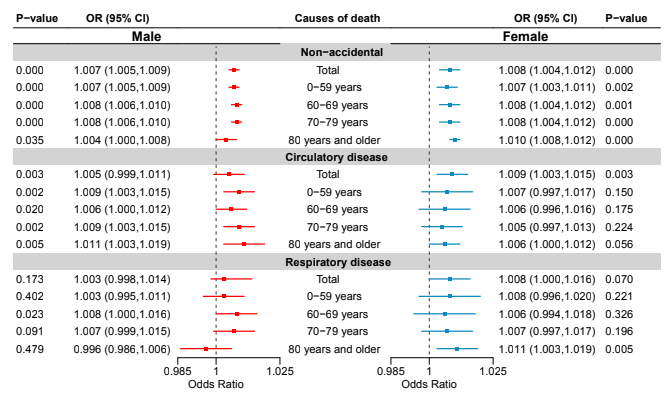


**Figure S2. Effect modification by district-level indicators in the PM_2.5_-mortality risk for each sex.** OR: odds ratio for a 10μg/m3 increase in PM_2.5_, P-value: P-value of OR (H_0_: OR is zero)

**
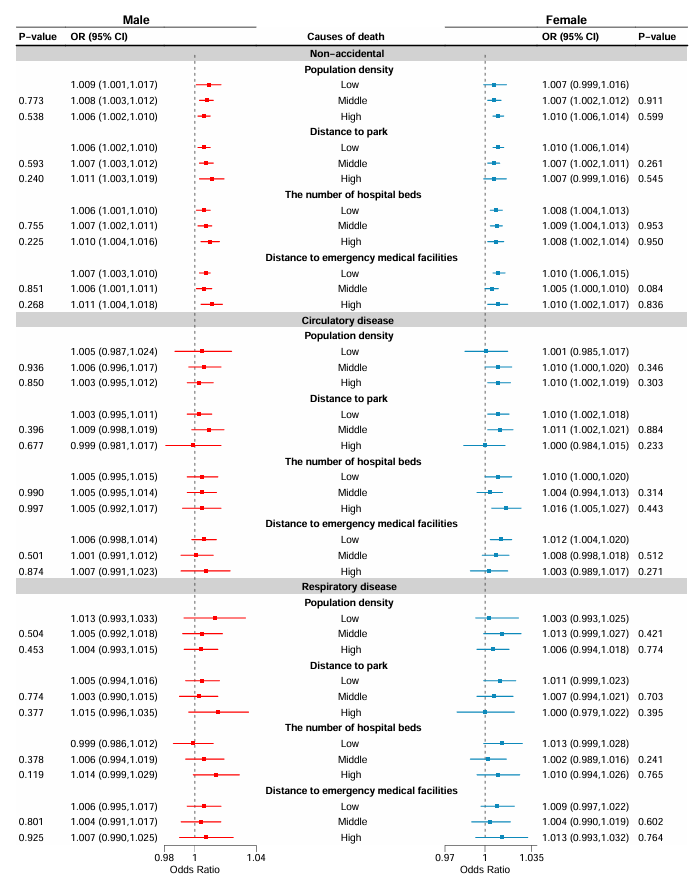
**

**References for the Supporting Information**

Byun, G., Kim, S., Choi, Y., Kim, A., Team, A.-C., Lee, J.-T., & Bell, M. L. (2024). Long-term exposure to PM2.5 and mortality in a national cohort in South Korea: effect modification by community deprivation, medical infrastructure, and greenness. *BMC Public Health*, *24*(1), 1266. <https://doi.org/10.1186/s12889-024-18752-y>

Di, Q., Amini, H., Shi, L., Kloog, I., Silvern, R., Kelly, J., Sabath, M. B., Choirat, C., Koutrakis, P., Lyapustin, A., Wang, Y., Mickley, L. J., & Schwartz, J. (2019). An ensemble-based model of PM2.5 concentration across the contiguous United States with high spatiotemporal resolution. *Environment international*, *130*, 104909. <https://doi.org/https://doi.org/10.1016/j.envint.2019.104909>

Gasparrini, A., Guo, Y., Hashizume, M., Lavigne, E., Zanobetti, A., Schwartz, J., Tobias, A., Tong, S., Rocklöv, J., Forsberg, B., Leone, M., De Sario, M., Bell, M. L., Guo, Y.-L. L., Wu, C.-f., Kan, H., Yi, S.-M., de Sousa Zanotti Stagliorio Coelho, M., Saldiva, P. H. N., Honda, Y., Kim, H., & Armstrong, B. (2015). Mortality risk attributable to high and low ambient temperature: a multicountry observational study. *The Lancet*, *386*(9991), 369-375. <https://doi.org/10.1016/S0140-6736(14)62114-0>

Kim, D., Jeong, J., Ko, Y., Kwon, Y., & Kim, Y. T. (2018). The construction of database of community health outcomes and health determinants in the Republic of Korea. *Public Health Wkly Rep KCDC*(11), 979-983.

Kim, H., Jang, H., Lee, W., Oh, J., Lee, J.-Y., Kim, M.-h., Lee, J. W., Kim, H. S., Kim, H., Lee, W., Kim, E., Kang, C., Song, I., Jang, H., Min, J., Kwon, D., Oh, J., Moon, J., Park, J., Kim, A., Ahn, S., Lee, J. H., & Ha, E.-H. (2024). Association between long-term PM2.5 exposure and risk of Kawasaki disease in children: A nationwide longitudinal cohort study. *Environmental Research*, *244*, 117823. <https://doi.org/https://doi.org/10.1016/j.envres.2023.117823>

Lee, W., Choi, M., Bell, M. L., Kang, C., Jang, J., Song, I., Kim, Y.-O., Ebi, K., & Kim, H. (2021). Effects of urbanization on vulnerability to heat-related mortality in urban and rural areas in South Korea: a nationwide district-level time-series study. *International Journal of Epidemiology*, *51*(1), 111-121. <https://doi.org/10.1093/ije/dyab148>

Lee, W., Prifti, K., Kim, H., Kim, E., Yang, J., Min, J., Park, J. Y., Kim, Y. C., Lee, J. P., & Bell, M. L. (2022). Short-term Exposure to Air Pollution and Attributable Risk of Kidney Diseases: A Nationwide Time-series Study. *Epidemiology*, *33*(1). <https://journals.lww.com/epidem/fulltext/2022/01000/short_term_exposure_to_air_pollution_and.3.aspx>

Park, J., Kang, C., Min, J., Kim, E., Song, I., Jang, H., Kwon, D., Oh, J., Moon, J., Kim, H., & Lee, W. (2023). Association of long-term exposure to air pollution with chronic sleep deprivation in South Korea: A community-level longitudinal study, 2008–2018. *Environmental Research*, *228*, 115812. <https://doi.org/https://doi.org/10.1016/j.envres.2023.115812>

Sun, Y., Milando, C. W., Spangler, K. R., Wei, Y., Schwartz, J., Dominici, F., Nori-Sarma, A., Sun, S., & Wellenius, G. A. (2024). Short term exposure to low level ambient fine particulate matter and natural cause, cardiovascular, and respiratory morbidity among US adults with health insurance: case time series study. *BMJ*, *384*, e076322. <https://doi.org/10.1136/bmj-2023-076322>

Wei, Y., Wang, Y., Wu, X., Di, Q., Shi, L., Koutrakis, P., Zanobetti, A., Dominici, F., & Schwartz, J. D. (2020). Causal Effects of Air Pollution on Mortality Rate in Massachusetts. *American Journal of Epidemiology*, *189*(11), 1316-1323. <https://doi.org/10.1093/aje/kwaa098>

Xu, S., Marcon, A., Bertelsen, R. J., Benediktsdottir, B., Brandt, J., Engemann, K., Frohn, L. M., Geels, C., Gislason, T., Heinrich, J., Holm, M., Janson, C., Markevych, I., Modig, L., Orru, H., Schlünssen, V., Sigsgaard, T., & Johannessen, A. (2023). Long-term exposure to low-level air pollution and greenness and mortality in Northern Europe. The Life-GAP project. *Environment International*, *181*, 108257. <https://doi.org/https://doi.org/10.1016/j.envint.2023.108257>
